# Mental Health Problems and Related Factors in Ecuadorian College Students

**DOI:** 10.3390/ijerph14050530

**Published:** 2017-05-15

**Authors:** Claudia Torres, Patricia Otero, Byron Bustamante, Vanessa Blanco, Olga Díaz, Fernando L. Vázquez

**Affiliations:** 1 Deparment of Psychology, Universidad Técnica Particular de Loja, Loja 11-01-608, Ecuador; bfbustamante@utpl.edu.ec; 2 Deparment of Psychology, University of A Coruña, 15071 A Coruña, Spain; patricia.otero.otero@udc.es; 3 Deparment of Evolutive and Educational Psychology, University of Santiago de Compostela, 15782 Santiago de Compostela, Spain; vanessa.blanco@usc.es; 4 Deparment of Clinical Psychology and Psychobiology, University of Santiago de Compostela, 15782 Santiago de Compostela, Spain; olga.diaz.fernandez@usc.es (O.D.); fernandolino.vazquez@usc.es (F.L.V.)

**Keywords:** mental health, prevalence, correlates, college students

## Abstract

Although the mental health problems of college students have been the subject of increasing research, there are no studies about its prevalence in Ecuadorian college students. The aim of this study was to determine the mental health problems and their associated factors in Ecuadorian freshmen university students. A sample of 1092 students (53.7% women; mean age = 18.3 years) were recruited from the Technical Particular University of Loja (Ecuador). Socio-demographic, academic, and clinical characteristics were gathered, as well as information on the participants’ mental health through a number of mental health screens. Prevalence of positive screens was 6.2% for prevalence of major depressive episodes, 0.02% for generalized anxiety disorders, 2.2% for panic disorders, 32.0% for eating disorders, 13.1% for suicidal risk. Mental health problems were significantly associated with sex, area of study, self-esteem, social support, personality and histories of mental health problems. The findings offer a starting point for identifying useful factors to target prevention and intervention strategies aimed at university students.

## 1. Introduction

The onset of most lifetime mental disorders occurs during young adulthood (late teens through early 20s) [1], and in many countries, the majority of young adults of this age are college students. In the U.S., for example, approximately 66% of the high school graduates enrolled in college in the subsequent school year and half of young adults attend college [2]. In developing countries, such as Ecuador, the number of young adults attending college is increasing [3]. More specifically, the Ecuadorian college population is growing annually by over 4%, with 39.6% of young adults attending college in 2012, and it is expected that this figure will exceed 50% by 2017 [4].

Previous research has demonstrated that the transition from high school to college can be stressful for students [5]. The college years represent a developmentally challenging transition to adulthood. University students, especially during the first year, are exposed to a variety of stressors that may trigger or exacerbate mental health problems, including not only academic burden but also those deriving from the change of environment, such as leaving the parental home, affective isolation, financial hardship, adaptation to methods of instruction very different from the high school or worries about the future [6,7]. These stressful experiences are related to an increased risk of psychopathology, which can have a negative impact for many aspects of well-being, including academic success [8] and future relationships [9].

Therefore, the mental health problems and overall well-being of college students have been the subject of increasing research. An elevated level of psychological distress and symptoms among college students has been found [10,11]. In fact, a prevalence of various mental disorders has been estimated as the following: between 5.3% and 17.3% for depression [12,13], between 1.6% and 7.0% for generalized anxiety disorder [12,14,15], between 0.6% and 4.1% for panic disorder [12,14,15], between 0.9% and 9.4% for eating disorders [15,16] and between 0.6% and 9.5% for suicidal ideation [12,13,17]. However, these studies on the prevalence of mental disorders did not focus on freshmen and most focused on anxiety disorders and depression [12,14]. There are few studies examining eating disorders, even though these usually appear in adolescence and early adulthood [18]. Furthermore, the majority of studies have a response rate of about 44–56.2% [12,16], which could introduce response bias.

The factors that influence the appearance of these mental health problems are as varied and numerous as they are complex. They are located at different levels (individual and environmental), can exert their influence directly or indirectly, and can maintain interconnections among themselves. Certain factors can influence the predisposition to suffer from a mental disorder, increasing the probability of onset or modifying a person’s response to some environmental hazard [19,20]. Therefore, psychological development must be understood as a complex process that responds to the influence of a multiplicity of factors closely linked to the environment or ecological context in which such development takes place. Specifically, in this study, we adopted as a theoretical framework the bioecological theory of Bronfenbrenner [21], later revised by Bronfenbrenner and Ceci [22] to analyze the mental health of the Ecuadorian university students from an integral, systemic and naturalistic perspective. This theory considers the development of the human being as a process that derives from the characteristics of individuals (including genetic and psychological) and of the context, both immediate and remote, as well as the relationships between them. These contexts are organized in systems called *microsystems* (immediate context of the individual that includes the individual conditions and the individuals or groups with which the individual has direct relationships), *mesosystem* (interconnections between microsystems), *exosystem* (connection between the immediate context of the individual and an environment in which the individual does not have an active role), and *macrosystem* (the culture in which the individual and his immediate contexts are immersed). This theoretical model is referred to as a Process–Person–Context–Time model. The critical element of this model is experience, which includes not only the objective properties, but also those that are subjectively experienced by the people living in that environment. Until now, it has been found that certain factors of microsystem and macrosystem such as gender and socioeconomic background influence the emergence of mental health problems in university students [23]. However, other psychological individual factors have been poorly investigated in students, even though they may also influence the predisposition to mental health problems. Among all of these, we selected self-esteem, social support, stressful life events and personality traits because they have been shown to be the main risk or protective factors for the development of depressive, anxiety and eating disorders as well as for suicidal behaviors in the general population [19,20,24]. 

Self-esteem can protect against mental health problems by reducing the stress [25] and the impact of negative thoughts [26] and has been related to depression, anxiety [27], eating disorders [28] and suicidal risk [29]. Social support provides instrumental, informational and emotional assistance that provide resources and can modulate the impact of negative life events [30] acting as a buffer for mental problems [31] and it has been related to depression [32], anxiety [33] and risk of suicide [34]. Stressful life events can increase the risk of suffering mental health problems because they pose a threat to the status of the individual and impose high adaptative demands [35]; they have been related to depression [36], eating disorders [37] and risk of suicide [38]. Personality traits condition the student’s way of acting, thinking and feeling, acting as triggers or moderators of mental health problems [39]; they have also been correlated to depression [40], anxiety [41], eating disorders [28] and suicidality [42]. In addition, there is little research on the specific factors of the academic context, such as the area of study. This factor determines the method of study, the demands of study, the academic climate, the projection of the future. Furthermore, it is known that different areas of study have different percentages of academic success and dropouts [43], all of which can influence the student emotional state, and if this is not handled properly, students could be predisposed to mental health problems [44,45]. Furthermore, no previous studies have analyzed the effect of associated factors simultaneously, which provides a more clinical, realistic and holistic perspective and considers the interconnections between the different contexts.

Finally, most of the studies were conducted in the United States [12,14,16,17]. To date, there are no studies about mental health problems in Ecuadorian college students, despite the fact that the students of this country have some peculiarities that distinguish them from those of other countries such as their Hispanic culture and the variety of ethnicities represented (including indigenous and montubios with little representation in the United States and Europe) and the predominance of mixed races [46]. Furthermore, it is known that the definitions of mental health problems, the forms of expression of such problems and the coping mechanisms employed can be significantly influenced by sociocultural factors [47,48]. It is therefore important to include aspects of Hispanic culture in the study of mental health problems, such as low income level, the importance of social and family support or ethnic diversity and mixed races, which can influence the prevalence of mental health problems [49,50]. In the general population, it was found that Hispanics have a significantly higher prevalence of affective disorders and comorbidity than non-Hispanic whites [51]. More specifically, among youngsters, Hispanic students were found to be more likely to suffer from depression than whites and multi-racial/ethnic students had more depression, suicidal ideation and self-harm than whites [12]. In addition, Ecuador is an emerging country that, with an increasing number of young adults attending college, represents the eighth largest economy of Latin America by GDP [52]. The purpose of this study was to determine the prevalence of major depressive episodes, generalized anxiety disorder, panic disorder, eating disorders and suicide risk, as well as associated risk factors, in freshmen at the Technical Particular University of Loja (Ecuador).

## 2. Materials and Methods

### 2.1. Sample

Participants were recruited from October 2012 to February 2013 from the student body of the Technical Particular University of Loja (Ecuador). Loja is a province in the south of Ecuador that covers an area of 11,027 km^2^ and has a population of 495,464 inhabitants, and has the second-highest percentage of college students, with a university attendance rate of 28.7% [53]. 

To participate in the study, students had to be enrolled in their freshman year and entering the university for the first time. Students who did not give their informed consent and upper-class students who had previously failed, and were thus repeating freshmen level courses, were excluded. The reason for this is that this study focused on freshman students entering the university for the first time, since there is evidence that they are exposed to a variety of new stressors related to the change of environment from secondary education to higher education that may trigger the development of mental disorders [7]. Students who have more than one year in college may have become familiar with and adapted to the new conditions of university life (acting as a confounding variable), so they were excluded from the study to avoid biasing the results. A total of 1113 registered students were invited to participate in the study during the 2012/2013 academic year.

To standardize the data collection process, a previously prepared assessment protocol was followed. Subsequently, we conducted a pilot test to refine the measuring instruments and improve the evaluation procedure. Five psychologists with more than five years of experience in teaching and research, who were previously trained, were involved in the data collection process. Training was given by two experts in psychological evaluation with more than nine years of experience and consisted of about eight hours of theoretical and practical workshops on the following: evaluation strategies, the evaluation protocol, instructions and correct completion of each instrument, frequent doubts that the students could have and how to address them, and role-playing of the application of assessment tools. The assessment was conducted in a classroom, in a collective and face-to-face format during non-examination periods for approximately one hour. During the evaluation, we first requested permission from the teacher who had been previously informed of the study and whose collaboration had been requested. Secondly, we made a presentation where we explained the nature of the study to the students, we gave out paper copies of the instruments, we provided instructions for their completion and addressed any questions raised by the students. Students self-administered the instruments and once all the instruments were completed by the students, they were collected. We then thanked the students for their participation in the study.

The participants read and signed their written consent prior to participation and their anonymity was guaranteed. Participation was voluntary and did not result in any academic, monetary, or other compensation. To minimize the loss of participants, we followed the strategies recommended by Hulley et al. for sample collection [54]; for example, made an appealing presentation of the study to the participants, emphasized the personal, social and scientific importance of their participation in the study, treated the patients with kindness, affection and respect, and avoided collecting information in an invasive and unpleasant manner. The study was carried out in accordance with the latest revision of the Declaration of Helsinki of 1975 revised in 2008, and was approved by the university Ethics Committee (Project identification code PY250-191-CEP-2011). The response rate was 99.7%. Three students (0.3%) declined to participate, stating that they did not feel comfortable answering that type of questions.

Of the students who participated (*n* = 1110), 18 were excluded because they were upper-class students that were re-taking freshmen courses. The final study sample was comprised of 1092 students (53.7% women, mean age 18.3 years) and characterized with respect to other variables in Table 1.

### 2.2. Instruments

Using an ad hoc questionnaire, data were collected on the participants’ sex, age, marital status, ethnicity, family monthly income, employment, area of study (assessing all study areas) and history of mental health problems, with the latter being defined as self-reported information on whether or not the student had suffered any mental health problems throughout their life.

Outcome measures were assessed using screening instruments. Depression was assessed using the *Patient Health Questionnaire-9* (PHQ-9 [55]; Spanish version of Díez-Quevedo et al. [56] validated in Spain), a self-administered nine-item questionnaire based on the DSM-IV criteria for major depressive disorder, the sensitivity and specificity of which was 84% and 92%, respectively. Generalized anxiety and panic disorder were assessed with the *Patient Health Questionnaire* (PHQ-A [55]; Spanish version of Díez-Quevedo et al. [56] validated in Spain), which is comprised of 15 basic questions based on the DSM-IV criteria with a sensitivity and specificity of 83% and 98%, respectively. Eating disorders were evaluated using the *Sick Control On Fast Food* (SCOFF [57]; Spanish version of García-Campayo et al. [58] validated in Spain), a self-administered five-item eating disorder screening questionnaire based on the DSM-IV. It has a 100% sensitivity and an 87.5% specificity for anorexia and bulimia. Suicide risk was evaluated with the *Suicidality Scale* (SC [59]; Spanish version of Salvo et al. [60]), a self-administered four-question scale with a scoring range of 0 to 12, in which the higher the score the greater the risk of suicide, and a cut-off point of 5, which has a sensitivity of 90% and a specificity of 79%.

Regarding possible associated factors, self-esteem was assessed using the *Rosenberg Self-Esteem Scale* (RSES [61]; Spanish version of Atienza et al. [62] validated in Spain). This scale is composed of 10 items; scores range from 10 to 40, with higher scores indicating higher self-esteem, with a Cronbach’s alpha of 0.92. Perceived social support was assessed with the *Multidimensional Scale of Perceived Social Support* (MSPSS [63]; Spanish version of Landeta and Calvete [64] validated in Spain), which includes 12 items with a scoring range of 1 to 7, in which a higher score indicates greater perceived social support, and has a Cronbach’s alpha of 0.88. Stressful life events were assessed with the *Social Readjustment Rating Scale* (SRRS [65]; Spanish version of Bruner et al. [66]), which includes 43 events for which the participant can indicate their occurrence (or not) and each event has a stress score from 11 to 100. The higher the score, the higher the stress level that the person has gone through in the last year. The test–retest reliability is 0.85 [67]. Personality characteristics were assessed with the *Eysenck Personality Questionnaire Revised-Abbreviated* (EPQR-A [68]; Spanish version of Sandín et al. [69] validated in Spain), which contains 24 items and four subscales (Neuroticism, Extraversion, Psychoticism and Sincerity). The neuroticism subscale refers to how easily and frequently a person becomes upset and anguished and it is related to emotional instability. The extraversion subscale refers to tendencies toward sociability, vivacity, activity and dominance. The psychoticism subscale implies a tendency towards psychological detachment from and a lack of concern for others. Lastly, the sincerity dimension evaluates the tendency to provide socially desired responses. Each subscale has a score range for each subscale between 0 and 6, with a higher score indicating greater presence of the characteristics (except for the sincerity subscale, where a higher score indicates lower sincerity). The Cronbach’s alpha for each subscale is the following: Neuroticism = 0.78, Extraversion = 0.74, Psychoticism = 0.63 and Sincerity = 0.54. 

### 2.3. Data Analysis

Statistical analyses were performed using the SPSS version 20.0 (IBM Corp., Armonk, NY, USA). The socio-demographic and academic characteristics of the participants were described with means and percentages, as appropriate. About 3.7% of the screening results for major depressive episode, about 2.0% for eating disorder and about 3.4% for suicide risk were eliminated from the analysis because the questionnaires were not filled out correctly. The chi-square test for qualitative variables or the Student *t*-test for quantitative variables were used to analyze whether there were significant differences between the excluded students and those included in the study for the sociodemographic, academic or clinical variables and for the prevalence of the mental health problems evaluated. To analyze whether there were differences between those who present mental health problems and those who did not present them according to sociodemographic, academic and clinical variables, bivariate analyses were carried out for qualitative variables through the Pearson's chi-square test or the Fisher’s exact test (with expected values less than 5) and for quantitative variables using the Student’s *t*-test for two independent samples with Bonferroni correction. Subsequently, logistic multivariate regressions analyses were performed to evaluate the association between current mental health problems and sociodemographic, academic and clinical variables that were previously significant in the bivariate analyses, in order to determine adjusted differences in associations between independent variables and mental health problems. The results are reported providing the adjusted odds ratio (OR) with 95% confidence interval (CI). Sociodemographic variables included sex, age, marital status, ethnicity, monthly family income, employment. Academic variable included area of study. Clinical variables included self-esteem, social support, stressful life events, personality traits and history of mental health problems.

## 3. Results

### 3.1. Participant Characteristics

Participants averaged 18.3 years of age (*SD* = 1.1), 53.7% were women, 98.5% were single, 95.1% were mestizos (i.e., persons with a mixed racial background), 44.0% had a monthly family income between $571 and $1040, 91.2% were not working, about 24.0% were taking courses in the life sciences area. Concerning clinical characteristics, the mean score for self-esteem was 31.7 (*SD* = 5.3), for social support was 53.0 (*SD* = 14.7) and for stressful life events was 190.1 (*SD* = 117.4). The mean score for the neuroticism subscale was 2.7 (*SD* = 1.8), 4.1 for extraversion (*SD* = 1.8), 1.0 for psychoticism (*SD* = 1.9), and 3.5 for sincerity (*SD* = 1.6). Finally, 91.5% had never experienced any mental health problems in the past (Table 1). 

We found no significant differences between the students excluded from the study and the final study sample regarding sociodemographic, academic and clinical variables.

### 3.2. Prevalence of Mental Health Problems

It was found that 6.2% (*n* = 65) of students met the criteria for diagnosis of a major depressive episode, 0.02% (*n* = 2) met criteria for generalized anxiety disorder, 2.2% (*n* = 24) for panic disorder, 32.0% (*n* = 343) were at risk of an eating disorder and 13.1% (*n* = 138) were at risk for suicide. 

We found no significant differences between the students excluded from the study and the final study sample in the prevalence of mental health problems.

### 3.3. Correlates of Mental Health Problems

The factors related to meeting criteria for depression were having lower self-esteem, *t*(1011) = 7.41, *p* < 0.001, less social support, *t*(1030) = 4.02, *p* < 0.001, greater neuroticism, *t*(73) = −9.00, *p* < 0.001, less extraversion, *t*(64) = 3.85, *p* < 0.001, and having a history of mental health problems, χ*^2^*(1, *N* = 1052) = 14.9, *p* < 0.001. The other sociodemographic, academic and clinical variables were not significant. When analyzed simultaneously (i.e., self-esteem, social support, neuroticism, extraversion, and history of mental health problems), it was found that students with higher self-esteem (adjusted OR = 0.91, 95% CI [0.87, 0.96]) are much less likely to have depression, and those with greater neuroticism (adjusted OR = 1.49, 95% CI [1.23, 1.80]) and a history of mental health problems (adjusted OR = 2.31; 95% CI [1.11, 4.82]) are more likely to have depression (Table 2).

Lower self-esteem, *t*(1046) = 4.25, *p* < 0.001, more stressful life events, *t*(1090) = 3.40, *p* = 0.001, lower neuroticism, *t*(969) = −4.65, *p* < 0.001 and having history of mental health problems, χ*^2^*(1, *N* = 1092) = 8.56, *p* = 0.003 were associated with meeting the criteria for panic disorder. The other socio-demographic, academic and clinical variables were not significant. When introducing all of these variables into the analysis, it was found that greater self-esteem (adjusted OR = 0.91, 95% CI [0.84, 0.99]) was associated with a lower risk of developing a panic disorder and greater neuroticism (adjusted OR = 1.89, 95% CI [1.33, 2.69]) was associated with a greater risk of developing it (Table 2). 

Being female, χ*^2^*(1, *N* = 1065) = 22.94, *p* < 0.001, enrolling in health sciences studies among all study areas, χ*^2^*(5, *N* = 1043) = 13.38, *p* = 0.02, lower self-esteem, *t*(1029) = 5.99, *p* < 0.001, more stressful life events, *t*(1068) = −3.93, *p* < 0.001, greater neuroticism, *t*(956) = −11.60, *p* < 0.001, and less extraversion, *t*(546) = 3.95, *p* < 0.001 were associated with risk of meeting criteria for an eating disorder. The other sociodemographic and clinical variables were not significant. When analyzing all variables simultaneously, it was found that having enrolled in health science majors and not in any other area of study (adjusted OR = 0.45, 95% CI [0.25, 0.81]) decreased the likelihood of having an eating disorder, while being female (adjusted OR = 1.55, 95% CI [1.13, 2.11]) and having greater neuroticism (adjusted OR = 1.49, 95% CI [1.36, 1.64]) increased the likelihood (Table 2).

Having lower self-esteem, *t*(1016) = 6.83, *p* < 0.001, lower social support, *t*(163) = 4.44, *p* < 0.001, greater neuroticism, *t*(182) = −9.93, *p* < 0.001, less extraversion, *t*(942) = 3.73, *p* < 0.001, and having a history of mental health problems, χ*^2^*(1, *N* = 1055) = 10.59, *p* = 0.001 were associated with a greater risk of suicide. The other sociodemographic and academic variables were not significant. When all variables were entered into the analysis, it was found that greater self-esteem (adjusted OR = 0.95, 95% CI [0.91, 0.99]) and greater perceived social support (adjusted OR = 0.98, 95% CI [0.97, 0.99]) decreased the likelihoods of suicide risk; however, greater neuroticism (adjusted OR = 1.51, 95% CI [1.33, 1.73]) increased it (Table 2).

## 4. Discussion

The purpose of this study was to examine mental health problems and associated factors in freshmen at the Technical Particular University of Loja (Ecuador). It was found that 6.2% of students met the criteria for diagnosis of a major depressive episode, which is consistent with the 5.3% in female students and 8.7% in students of both sexes identified as having a major depressive episode by Vázquez et al. [15] and Vázquez and Blanco [13], although it was less than the 17.3% found by Eisenberg et al. [12]. However, these differences are reduced if we consider that this study, unlike the previous ones, focused only on freshmen. Self-esteem was a protective factor against meeting the criteria for major depressive episode; however, neuroticism and having a history of mental health problems were risk factors. This result is consistent with the findings in the scientific literature. Specifically, at universities, Song et al. [70] found that low self-esteem, concern over mistakes and high neuroticism were associated with depressive symptoms. One possible explanation is that the characteristics of neuroticism such as emotional instability, insecurity, tendency toward guilt and somatization are usually present in depressive disorders. Likewise, having a personal history of mental health problems (especially depressive symptoms, anxiety and substance abuse) has been considered a factor that increases the risk of developing depression [71,72].

It was also found that 0.02% met the criteria for generalized anxiety disorder. This prevalence is much lower than found in other studies [12,14,15]. Given the low prevalence, we have been unable to analyze what variables may be associated with this mental disorder. One hypothesis for this low percentage could be that the majority of students who attend this university come from the city of Loja and, therefore, they have no concerns related to new situations that generate uncertainty due to leaving the family home and going to live in other parts of the country, such as looking for housing or economic problems. 

Furthermore, it was found that about 2.2% of students met the criteria for panic disorder. This data is between the 0.6% prevalence found by Vázquez et al. [15] for panic disorder with and without agoraphobia and the 4.1% found by Eisenberg et al. [12]. Having high self-esteem is a protective factor against panic disorder, in line with the evidence that highly frequent attacks were predicted by low self-esteem [73]. Furthermore, high neuroticism was a risk factor for development of the disorder. This result is consistent with the previous findings in clinical samples, where significantly higher neuroticism scores were found in panic disorder patients compared to the control group [41].

On the other hand, it was found that 32.0% of students had a risk of suffering from an eating disorder, which is higher than the 0.9% to 9.4% prevalence found in prior studies with college students [15,16]. Given that the age of highest risk of onset of anorexia and bulimia is 15 to 19 years old [23], our greater prevalence rate may be due to the age of our students (only freshmen 17 to 24 years old), compared to the 21.9% of students from various years of college over 31 years of age in the study of Eisenberg et al. [16]. Among all areas of study analyzed, belonging to the health sciences was a protective factor against these disorders. Although research in relation to the variable area of study is limited, one possible explanation for this relationship is that health science (medicine and psychology) students have knowledge about the risks and consequences of these disorders, which may be a protective factor against them. Conversely, female gender and neuroticism act as risk factors for these disorders. The gender gap might be influenced by maintaining the standards of beauty associated with thinness for females and the vulnerability of the stage of development of the participants in this study, and is consistent with numerous studies worldwide that have found that these disorders occur most frequently in the young female population [23]. Furthermore, the relationship found between high neuroticism and risk for eating disorders is consistent with the findings that female college students with eating disorder symptoms differed from those who were asymptomatic in neuroticism, extraversion and agreeableness [74].

Finally, about 13.1% of the college students were identified as being at risk for suicide. These findings have a high clinical and social relevance, because suicide is the second-leading cause of death in those 15 to 29 years of age worldwide [75]. This prevalence is greater than the 0.6% and 9.5% for suicidal ideation found in previous studies [12,13,17], which may be due to the different assessment tools used, as only this study had a validated tool. It was found that self-esteem and social support were protective factors against suicide risk, in line with previous findings. Self-esteem is negatively associated with suicidal ideation even after controlling for depression and hopelessness in psychiatric patients [29]. In addition, it has been seen that feelings of loneliness and not belonging and not having support from people around them, especially at this stage of development, may predispose young adult college students to experience emotional destabilization and lead to extreme situations such as suicide [34]. Moreover, neuroticism as a personality trait acted as a risk factor for suicide risk. This personality trait, frequently associated with negative affectivity and maladaptive coping strategies, has consistently been found to be associated with increased suicidality [42].

The present prevalence of mental disorders among our university students was higher than those observed in community studies, such as the prevalence of 4.4% for major depression and 0.6% for panic disorder found in Latin America [76] or the 12-month prevalence of 3.6% for major depression, 2.7% for panic disorder and 1.5% for any eating disorder observed among 14- to 24-year-old people [77], which is consistent with the greater risk of psychopathology of which college students are exposed. 

However, this study should be interpreted within the scope of their limitations. This is a cross-sectional study and, therefore, the relationships analyzed cannot demonstrate causality. Moreover, Spanish versions of the questionnaires were not validated in Ecuador. As it is a study with self-reported questionnaires, there is the possibility of response bias. Future research with hetero-administered tools and structured clinical interviews could help to contrast the information obtained. Given that our sample is limited to students from a single university, the results may not be generalizable to students located at other universities or countries; however, this is unlikely to be the case, because the distribution by age, sex or areas of study is similar to the universities in the rest of the country. 

## 5. Conclusions

This is the first mental health problems study of Ecuadorian college students where associated risk and protective factors were also identified. It provides clear indicators about the mental health needs of the university population and its results will allow research priorities to be organized. Since the majority of these disorders can be treated effectively through evidence-based psychological and/or psychopharmacological methods, it is recommended that treatment and prevention interventions be applied [78,79]. Specifically, promoting self-esteem, social support and extraversion (e.g., training social skills), as well as strategies for management of neuroticism by clinicians and teachers. Universities are in fact in an excellent position to promote those among young people, providing them not only with academic services but also with residences, social environment, extracurricular activities and health services.

## Figures and Tables

**Table 1:** Socio-demographic, academic and clinical characteristics.

Variables	*n*	%
Sex		
Male	506	46.3
Female	586	53.7
Age		
* M*	18.3	
* SD*	1.1	
Range	17–24	
Marital status		
Single	1076	98.5
Married	7	0.6
Widow/Widower	1	0.1
Divorced	1	0.1
Domestic partnership	7	0.7
Ethnicity		
Mestizo	1039	95.1
White	35	3.2
Afro-Ecuadorian	10	0.9
Montubio	2	0.2
Indigenous	5	0.5
Others	1	0.1
Monthly income ($)		
0 to 570	229	21.0
571 to 1040	481	44.0
1041 to 1610	218	20.0
1611 to 2180	119	10.9
>2181	45	4.1
Works		
No	996	91.2
Yes	96	8.8
Area of study		
Legal and social sciences	123	11.3
Economic sciences	212	19.4
Arts and humanities	92	8.4
Life sciences	262	24.0
Technological sciences	241	22.1
Health sciences	162	14.8
Self-esteem		
*M (SD)*	31.7 (5.3)	
Social support		
*M (SD)*	4.4 (1.2)	
Stressful life events		
*M (SD)*	190.1 (117.4)	
Personality		
Neuroticism, *M (SD)*	2.7 (1.8)	
Extraversion, *M (SD)*	4.1 (1.8)	
Psychoticism, *M (SD)*	1.0 (1.9)	
Sincerity, *M (SD)*	3.5 (1.6)	
History of mental health problems		
No	999	91.5
Yes	93	8.5

**Table 2:** Correlates of mental health problems.

Mental Health Problem	Adjusted OR	95% CI	
		Lower Limit	Upper Limit
Major depressive episode			
Self-esteem	0.91	0.87	0.96
Social support	0.98	0.97	1.00
Neuroticism	1.49	1.23	1.80
Extraversion	0.91	0.78	1.06
History of mental health problems			
No	1.0		
Yes	2.31	1.11	4.82
Panic disorder			
Self-esteem	0.91	0.84	0.99
Stressful life events	1.01	1.00	1.01
Neuroticism	1.89	1.33	2.69
History of mental health problems			
No	1.0		
Yes	2.79	0.94	8.29
Eating disorders			
Sex			
Male	1.0		
Female	1.55	1.13	2.11
Area of study			
Legal and social sciences	1.0		
Economic sciences	0.86	0.50	1.46
Arts and humanities	0.56	0.28	1.11
Life sciences	0.67	0.39	1.14
Technological sciences	0.69	0.40	1.18
Health sciences	0.45	0.25	0.81
Self-esteem	0.97	0.94	1.00
Stressful life events	1.04	1.00	1.08
Neuroticism	1.49	1.36	1.64
Extraversion	0.97	0.89	1.06
Suicide risk			
Self-esteem	0.95	0.91	0.99
Social support	0.98	0.97	0.99
Neuroticism	1.51	1.33	1.73
Extraversion	1.02	0.91	1.14
History of mental health problems			
No	1.0		
Yes	1.64	0.89	3.03

Note: OR = Odds Ratio; CI = Confidence interval.
